# Supplementary material for: Adaptive multi-objective control explains how humans make lateral maneuvers while walking
Source: PLoS Comput Biol. 2022 Nov 14;18(11):e1010035. doi: 10.1371/journal.pcbi.1010035 (PMC9704766; doi:10.1371/journal.pcbi.1010035)
Supplement: S1 Text — We show that zB is a reasonable proxy of lateral position of the body’s CoM at each step. (PDF) [file pcbi.1010035.s001.pdf]

Adaptive Multi-Objective Control Explains How Humans Make Lateral Maneuvers While Walking

David M. Desmet Joseph P. Cusumano Jonathan B. Dingwell

PLoS Computational Biology

SUPPLEMENTARY TEXT #S1: Relation of Body Position (z_B) to Center-of-Mass (CoM)

We define lateral body position (z_B) at each step as the midpoint between consecutive heel strikes ([1]; Fig. 1C). In continuous time, the center-of-mass (CoM) oscillates between the feet as each new step is taken [2, 3]. Near the moment of heel strike, the CoM passes approximately across the midline between the feet, during both straight walking ([3, 4]; Fig. S1A) and when performing lateral maneuvers [5]. In our work, we analyze walking *step-to-step*: each step is treated as a single event. Fig. S1A shows, however, that no single value of CoM value can easily be assigned to a complete step. However, we can take z_B as a *proxy* of a discrete (once per step) estimate of the lateral CoM location for each step (Fig. 1B).

To see this, we computed continuous-time lateral CoM trajectories from pelvis centroid motion [6] (Fig. S1B). We then correlated the lateral CoM values that occurred at each heel strike with z_B for that step (Fig. S1C). In all cases, z_B was very strongly correlated with these CoM values, for steady straight-ahead walking ($R^2 = 0.82$) and also for each of the preparatory ($R^2 = 0.81$), transition ($R^2 = 0.76$), and recovery ($R^2 = 0.82$) steps taken during the lane-change maneuver.

These data confirm that z_B is a reasonable proxy that represents a discrete (i.e., once per step) measure of the approximate lateral position of the body's center-of-mass (CoM) at each step.

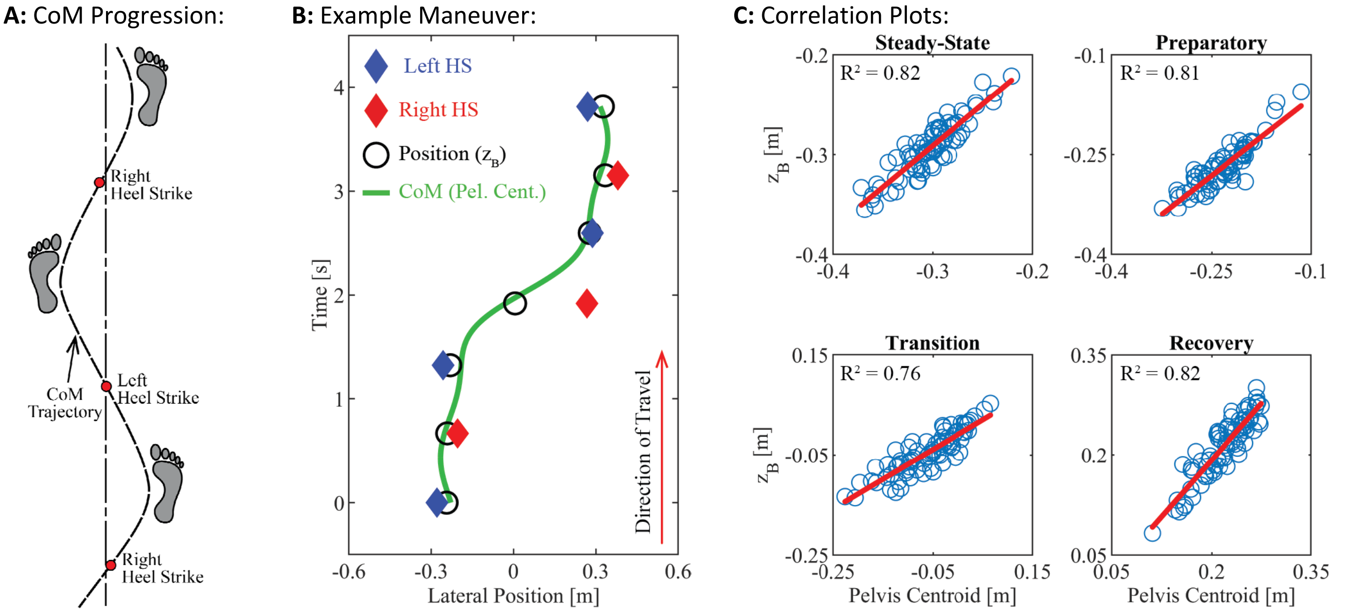

Figure S1: A: Schematic of foot placements and center-of-mass (CoM) trajectory during typical straight-ahead walking, with instances of left and right heel strikes indicated (see also [3], Fig. 15 and [4], Fig. 2). **B:** Data for Left (♦) & Right (♦) steps, lateral position (z_B) locations (○), and CoM (pelvis centroid) trajectory (—) for a typical lane-change trial from this experiment (similar trends shown in [5], Fig. 2). **C:** Correlations of body CoM at each heel strike vs. z_B. Note: All $R^2 \geq 0.76$.

References:

1. Dingwell JB, Cusumano JP. Humans Use Multi-Objective Control to Regulate Lateral Foot Placement When Walking. PLoS Comput Biol. 2019;15(3):e1006850. doi: https://doi.org/10.1371/journal.pcbi.1006850.
2. Bruijn SM, van Dieën JH. Control of human gait stability through foot placement. J R Soc Interface. 2018;15(143):1-11. doi: https://doi.org/10.1098/rsif.2017.0816.
3. Winter DA. Human Balance And Posture Control During Standing And Walking. Gait Posture. 1995;3(4):193-214. doi: https://doi.org/10.1016/0966-6362(96)82849-9.
4. Orendurff MS, Segal AD, Klute GK, Berge JS, Rohr ES, Kadel NJ. The effect of walking speed on center of mass displacement. J Rehabil Res Develop. 2004;41(6):829-34. doi: https://doi.org/10.1682/jrrd.2003.10.0150.
5. Wu M, Matsubara JH, Gordon KE. General and Specific Strategies Used to Facilitate Locomotor Maneuvers. PLoS ONE. 2015;10(7):e0132707. doi: http://dx.doi.org/10.1371/journal.pone.0132707.
6. Havens KL, Mukherjee T, Finley JM. Analysis of biases in dynamic margins of stability introduced by the use of simplified center of mass estimates during walking and turning. Gait Posture. 2018;59:162-7. doi: https://doi.org/10.1016/j.gaitpost.2017.10.002.
